# Widening access in the European Union Higher Education Arena: Introducing Neurotech^EU^

**DOI:** 10.25122/jml-2021-1002

**Published:** 2021

**Authors:** Dafin Muresanu, Stefan Strilciuc, Anca Dana Buzoianu

**Affiliations:** 1. Iuliu Hatieganu University of Medicine and Pharmacy, Cluj-Napoca, Romania; 2. RoNeuro Institute for Neurological Research and Diagnostic, Cluj-Napoca, Romania

Neurotech^EU^ – the European University of Brain and Technology (www.theneurotech.eu) brings together eight leading universities across the four corners of Europe, 250+ associates, including previously funded European Universities, partner research institutions, companies, societal stakeholders, cities, and (non) governmental organizations to implement a comprehensive training program for all segments of society and in all regions of Europe. The mission of Neurotech^EU^ is to build a trans-European network of excellence in brain research and technologies to increase the competitiveness of European education, research, economy and society.

The foundation phase of Neurotech^EU^ will include five key deliverables, as described by the European Commission factsheet (https://ec.europa.eu/education/sites/default/files/document-library-docs/european-universities-factsheet-neurotech-eu.pdf):

• **Neurotech^EU^ Campus+** will create the crucial shared virtual space, an extension of the partnering organizations, where students, teachers, and administrators work together without administrative, cultural and societal obstacles to provide physical, digital and blended education;
• **Neurotech^EU^ Graduate School** will provide co-tutelage education at the master's and doctoral level to train top-flight researchers in a multidisciplinary and intersectoral setting. It will promote innovation and an entrepreneurial mindset. Each student will work on a **Neurochallenge**, i.e. societal challenge that can be met by neuroscientific and neurotechnological knowledge and solutions;
• **Neurotech^EU^ Life-long Learning Centre** will support the continued training of its graduates and society at large. It will provide the necessary knowledge, skill sets, and competencies for individuals to adapt to the changing personal, civic, societal and employment-related needs and provide them opportunities in brain research and technologies;
• **Neurotech^EU^ Spaces** will be the virtual collaboration platform. Based on a suite of open-source software, the Spaces will provide the necessary tools to communicate, create, share, and store information safely. It will address users' needs from any background, from a scholar studying the philosophy of mind to students jointly working on robotic control algorithms;
• **Neurotech^EU^ Ecosystem**. Modern universities need to be integrated into society and the economy. This integration will help universities focus on their education, talent development and innovation efforts based on the current and future needs of the world. It will also ensure that their graduates are embedded in an ecosystem, boosting their employability and promoting entrepreneurship. Neurotech^EU^ will form its ecosystem by synergizing stakeholders in society, education, research and innovation sectors across the continent.

The “Iuliu Hațieganu” University of Medicine and Pharmacy from Cluj-Napoca, Romania, one of its eight founders, coordinates Alliance efforts for *Widening access – diversity, multilingualism and multiculturalism*. “Iuliu Hațieganu” University of Medicine and Pharmacy from Cluj-Napoca develops its educational programmes in three languages (Romanian, French and English). Combined with an organizational approach that promotes internationalization, the university has created a diverse, multilingual and multicultural education environment with the highest number of international students in Romania. By pooling experience across consortium partners, the Neurotech^EU^ Alliance aims to integrate international students while fostering diversity, equality and inclusion.

Despite a sustained push over the last decades for academia to become more accessible for everyone, disparities in higher education are ever-present. Only a fraction of female students choose science, technology, engineering, and mathematics (STEM) related fields. In 2018, only 3% of students joining information and communication technology courses worldwide were women. Women are more attracted to STEM courses in some world regions than others, but the global situation remains characterized by stark gender imbalances. Income inequality is another issue with regard to access to education. Individuals from lower socioeconomic backgrounds face discrimination in many aspects of their life, including education, as financial barriers leave students struggling to pursue their goals. Moreover, people living with disabilities also face injustice and discrimination in higher education. Driven by the issues mentioned earlier and many others to be identified at the Alliance level, we are keen to discover and disseminate best practices for promoting equality and diversity. Moreover, we are looking to identify and develop practical learning tools to support our shared vision. Existing and novel virtual and blended learning are emerging as primary educational delivery paradigms, promising many opportunities for students and teachers alike by providing access and higher curriculum flexibility despite economic, geographical limitations and, more recently, epidemiological conditions.

In the coming months, our Alliance will publish a report summarizing best practices available in the literature as an initial step in laying the foundation of Neurotech^EU^. Based on an initial exploration of information sources, there is insufficient available research focused on vulnerable but overlooked groups, such as mature students, carers and care leavers, ethnic minority students and vocational students. Therefore, it will be essential to integrate case studies from partner universities, showcasing real-world experiences that may help build a cohesive approach in the Alliance.

Neurotech^EU^ must now deploy efforts to define vulnerable populations, map barriers of access to education, and research capacity must be prioritized to support the development of a progressive strategy of implementation of all feasible and desired best practices for widening access, as to promote effective use on available time and resources in the consortium. Universities must identify surveillance mechanisms for issues related to widening access and participation to education, following students early (i.e., starting at high-school level) and longitudinally across their educational and career track. This step is essential to evaluate interventions in this broad field, opening universities to evidence-based decision-making rather than anecdotal reports or subjective input from students and staff.

With all these considerations in mind, Neurotech^EU^ is genuinely committed to creating an inclusive environment based on diversity, innovation, connection and inspiration and work together to build the future of Higher Education. Stay tuned for invited editorials related to experiences with widening access to higher education from Neurotech^EU^ Alliance partners in the coming issues of Journal of Medicine and Life!

